# Contralateral Posterior Chamber Phakic Intraocular Lens Implantation as Rehabilitation of Refractive Lens Exchange with a Monofocal Intraocular Lens in a Young, Nonpresbyopic, Bilateral Highly-Myopic Patient

**DOI:** 10.1155/2019/8791071

**Published:** 2019-10-16

**Authors:** Kepa Balparda, Claudia Marcela Vanegas-Ramírez, Laura Segura-Muñoz, Manuela Gómez-Londoño

**Affiliations:** ^1^Department of Cornea and Refractive Surgery, Clínica de Oftalmología Sandiego, Medellín, Colombia; ^2^Department of Ophthalmology, Universidad Pontificia Bolivariana, Medellín, Colombia; ^3^School of Medicine, Universidad Pontificia Bolivariana, Medellín, Colombia

## Abstract

**Background:**

Refractive errors are widespread in the human population; nowadays, numerous surgical options allow for efficient and safe correction them. One of the main elements to ensure success in this kind of intervention will depend on the careful patient and surgical approach selection. Excimer laser corneal surgery is considered by most for low to moderate ametropias. Another option, which has been suggested may be safer, is to cut a small corneal lenticule with femtosecond laser, and then extracting it through a small incision. Nevertheless, in some specific cases, such as patients with high refractive error or those with some corneal abnormality, laser corneal ablations are considered unsafe from either a biomechanical or refractive standpoint. In this kind of particular cases, Phakic Intraocular Lens (P-IOL) implantation constitutes attractive, highly predictable and safe option.

**Objective:**

The authors want to show the case of a young high-myopic woman, already pseudophakic in one eye, where the P-IOL implantation in the fellow eye yielded excellent short-term visual results, and high patient's satisfaction, is presented.

**Materials:**

The authors present the case of a 32-years-old, highly myopic female patient underwent a Refractive Lens Exchange (RLE) with a monofocal Intraocular Lens (IOL) implantation in her left eye elsewhere, and developed severe visual issues, especially regarding near-work. Symptoms resolved through the implantation of a posterior chamber P-IOL in the contralateral eye.

**Results:**

The postoperative course was unremarkable, inflammation was mild, and visual recovery was quick. There was no need to perform any procedure on her left eye or to use any reading glasses, as unilateral effective near vision through her right eye was enough for all her daily tasks.

**Conclusions:**

RLE in young pre-presbyopic highly myopic patients may not be an advisable alternative in most cases, because of the high risks of retinal complications observed. In addition, eliminating accommodation will cause significant limitations, and multifocal IOLs currently available are far from the quality of vision that a young human crystalline lens yields. On the other hand, implantation of a P-IOL is a good option if eye conditions are optimal, as it preserves natural accommodation. In this case an EyeCryl Phakic Toric® IOL showed excellent short-term refractive predictability and safety.

## 1. Introduction

Refractive errors are widespread in the human population. According to Holden and collaborators [1], 22.9% of the world population (1.406 million people) have myopia [defined as a refractive error ≤ −0.50 Diopters (D)], while 163 million (2.7% of the world population) have high myopia (refractive error ≤ −5.0 D). Among some populations in Southeast Asia, the prevalence of myopia reaches much higher levels in young adults, being up to 80–90%, while high myopia prevalence could reach 20% and they have increased during the last decades, conforming a true epidemic of myopia [2]. In Colombia data are still not so high. Recently it was found that 11.6% of children (8–17 years old) and 14.4% of adults (35–55 years old) were myopic [3].

Uncorrected ametropia may significantly decrease quality of life, and surgical correction may have the advantage of setting patients free from external refractive elements for most situations. Nowadays, numerous surgical options allow for efficient and safe correction of refractive errors. One of the main elements to ensure success in this kind of intervention will depend on the careful patient and surgical approach selection, depending on each case. Excimer laser corneal surgery (including LASIK, PRK, and other similar surgeries) is considered by most as the go-to surgical technique for low to moderate ametropias [4]. Another option, which has been suggested may be safer, is to cut a small corneal lenticule with femtosecond laser, and then extracting it through a small incision (Relex SMILE).

Nevertheless, in some specific cases, such as patients with high refractive error or those with some corneal abnormality (thin pachymetry or ectasias), laser corneal ablations are considered unsafe from either a biomechanical (risk of ectasia) or refractive (excessive corneal flattening) standpoint. In this kind of particular cases, Phakic Intraocular Lens (P-IOL) implantation constitutes an attractive, highly predictable and safe option. The advantages of P-IOLs over pseudophakic IOLs include that the former allow for keeping the accommodation potential of the crystalline lens in young patients, while the use of pseudophakic IOLs eliminates it, and also that they are not associated with retinal complications in high myopes. On the other hand, phacoemulsication has been related with significant increased risk of retinal detachment in such patients [5–7].

In this article, the authors present the case of a 32-years-old, highly myopic female patient, who underwent Refractive Lens Exchange (RLE) with a monofocal IOL implantation in her left eye at another clinic. The patient then developed severe visual issues, especially regarding near-work vision. Symptoms resolved through the implantation of a P-IOL in the contralateral eye.

## 2. Case Presentation

A 32-years-old female patient concurred to the main author's private practice in the search for a second opinion regarding her ocular state. The patient had undergone RLE with the implantation of a monofocal IOL (AcrySof SA60AT; Alcon Surgical, Forth Worth, United States) in her left eye seven months before. From the moment of surgery, the patient developed severe asthenopia, headache, and dizziness, especially for near vision. Due to her job as a secretary, she was an avid computer user during at least 9 hours per day. The patient did not tolerate the use of contact lenses, and she was also unable to use glasses comfortably. The patient ended up resigning her job due to her inability to work comfortably on the computer.

Upon questioning about this medical decision, she claimed that she had been referred to a Comprehensive Ophthalmology specialist through her public health insurance because of high bilateral myopia. She also claimed that the original Surgeon explained that the Colombian public health system did not include P-IOL implantation, so he advised to patient to undergo RLE in both eyes and then use reading glasses. Reviewing previous clinical records, she was found to have original myopia of −18.00 D/+1.50 D × 140° in her left eye, with no history of congenital nor juvenile cataract. She also referred that the Surgeon never suggested using any high-technology IOL (trifocal, multifocal, etc.). Prior to her original surgery, she used glasses, as she did not tolerate contact lens use.

Upon clinical evaluation, the patient had an uncorrected distance visual acuity of 20/2000 and 20/40 in her right and left eyes, respectively. Her manifest refraction and distance corrected visual acuity were −22.00 D/+4.75 D × 105° (20/20) on her right eye and −0.75 D/+1.00 D × 137° (20/25) on her left eye. Near uncorrected vision on her right eye was 20/20 while it was 20/400 on the left one.

The physical examination of the anterior segment of her right eye was unremarkable. Her retina was thin upon examination, but not predisposing lesions were detected. Her left eye had a clear cornea, with a Monofocal IOL well-placed inside the capsular bag and no posterior capsular opacification. The rest of the clinical evaluation was completely normal.

Ocular biometry of the right eye (IOLMaster 500; Carl Zeiss Meditec AG; Jena, Alemania) reflected a flat keratometry of 41.9 D × 17°, and a steep keratometry of 45.1 D × 107°; her white to white distance was 11.2 mm, and her anterior chamber depth was 3.06 mm. Her endothelial cell count of the right eye was 2565 cells/mm^2^ with all parameters being normal. In an effort to normalize her vision and diminish her near-vision symptoms, especially since she was unable to tolerate either glasses or contact lenses, we suggested the patient undergo posterior chamber P-IOL implantation in her right eye and leave the left eye untouched. She accepted and a −21.50 D/+3.50 D × 90° (size 12.0 mm) posterior chamber Toric P-IOL (EyeCryl Phakic IOL, Biotech Vision Care; Ahmedabad, India) was implanted in her right eye. The refractive target was +0.50 D.

Surgical implantation was performed under peribulbar anesthesia by the main author (K.B.) as follows: the patient was draped and the eye cleaned; then a paracenthesis and a 2.8 mm main incision were created, and the anterior chamber was filled with 1% Sodium Hyaluronate Ophthalmic Viscosurgical Device (Provisc®; Alcon Surgical; Forth Worth, United States). The P-IOL was mounted and injected inside the anterior chamber. Afterward, the lens was positioned in the correct toric markings, and all four haptics were positioned behind the iris in the ciliary sulcus. Then, the Ophthalmic Viscosurgical Device was removed, and 1% Acetylcholine was injected intracamerally to achieve proper pupillary miosis. Post-surgical regimen included 0.5% Moxifloxacin (Moxipharm®; Opharm; Bogotá, Colombia) and 1% Prednisolone (Predilab®; iLab Colombia; Bogotá, Colombia).

The postoperative course was unremarkable, inflammation was mild, and visual recovery was quick. On the first day after surgery, her uncorrected distance visual acuity of 20/25 and uncorrected near visual acuity of 20/20 in her right eye. On the first month after surgery, her uncorrected distance visual acuity was 20/15 in her right eye, with a manifest refraction of +0.75 D/−0.25 D × 74°. The patient claimed that starting at two days after surgery, her visual symptoms resolved about 100%, and she was able to use computed and write normally. Headaches subsided, and she was able to return to her job. Her endothelial cell count in the right eye, one month after surgery was 2511 cells/mm^2^and no significant change on cellular size or form could be detected.

So far, the patient has been observed for four months after surgery. On last evaluation, her uncorrected distance and near vision have remained unchanged, and her last manifest refraction was +0.75 D/−0.25 D × 78°.

There was no need to perform any procedure on her left eye or to use any reading glasses, as unilateral effective near vision through her right eye was enough for all her daily tasks.

## 3. Discussion

High myopia, usually defined as a spherical equivalent ≤ −5.00 D or ≤ −6.00 D is a relatively common condition worldwide, with wide geographical variance [1, 2, 8]. As these patients frequently look for refractive corrective procedures, it is the responsibility of the Refractive Surgeon to determine the best option for them, and this decision must be based on several elements that are both clinical and anatomical. Patients with low to moderate myopia and suitable cornea may be good candidates for laser approach, either excimer one (LASIK, PRK, etc.) or Femtosecond-based one (SMILE) [9]. Those patients with relatively low refractive error and some corneal irregularity, such as those with mild keratoconus may undergo combined surgery with topography-guided PRK and Corneal Collagen Crosslinking (CXL); technique known as Athen's Protocol [10, 11].

Nevertheless, patients with high myopia or those with abnormally thin cornea represent a complex clinical scenario, as corneal-based surgery would create undesirably unstable anterior segment with potential complications such as laser-related ectasia. In these patients, IOL implantation may be considered, this will provide mean to correct almost any value of refractive error while preserving full corneal stability. The decision on whether to maintain or extract the crystalline lens will reside many elements, including the patient's age and lens status, whether it is clear or clouded by cataract.

Patients with visually-significant cataract do well with crystalline lens extraction via phacoemulsification coupled with a pseudophakic IOL implantation, either monofocal or multifocal/trifocal/EDOF, keeping in mind that the former group will need the help of reading glasses for most near-vision activities [12]. Presbyopic patients with clear lens, especially those over 55 years old could be good candidates for RLE with implantation of pseudophakic IOL, most probably a multifocal/trifocal/EDOF. In some of them, it may be acceptable to implant a monofocal IOL keeping in mind that these will not completely compensate for presbyopia unless some monovision is attempted and achieved.

All these options are applicable in patients who are already presbyopic, especially those over 50–55 years old.

Nevertheless, nonpresbyopic patients represent exceptional group that demands different approach. Patients under the age of 40–45 still have useful accommodative potential, their near-vision will be clear as long as the eye is emmetrope, or near so. Therefore, in young patients, preserving the integrity of the crystalline lens is of paramount importance, and proper technique should be sought, as long as it is viable according to the clinical scenario.

Furthermore, lens extraction has been related with increased risk of retinal detachment in young highly myopic patients. In a classic study was related with late medium-term (7 years) prevalence of retinal detachment of 8.1% in pre-presbyopic patients [5]. In a more recent study conducted by Alio et al. [6], patients were divided into 2 groups according to age (group 1 ≤ 50 years and group 2 > 50 years) and axial length (≤ 28.0 mm and > 28.0 mm). Eyes with longer axial lengths showed greater incidences of retinal detachment. This complication was also more frequent in younger patients (3.65% in group 1 compared to 2.52% in group 2, *p* < 0.05). Risk factors for retinal detachment included: increase in axial length, age less than 50 years, males, sex, Caucasian race, peripheral retinal degenerations, intraoperative rupture of the posterior capsule, and Nd: YAG capsulotomy. Therefore, currently it is considered that RLE should not be performed in young eyes with no posterior vitreous detachment [7].

Some authors have suggested that an RLE with a multifocal IOL implantation may be a viable option for young, nonpresbyopic patients not good candidates for any other kind of approach [13]. Frings et al. [14] described their experience with 16 patients (5 myopic and 11 hyperopic) with mean age of 31 ± 6 years old who were implanted with a multifocal IOL. Both objective and subjective results were excellent, and only one of the patients “would not have chosen this surgery again” [14]. This may be viable option in highly hyperopic young patients with shallow anterior chamber depth that contraindicates P-IOL implantation, but this usually is not the case for highly myopic patients (such as the one we report here) whose eyes (including anterior chamber depth) tend to be bigger than average.

As has been mentioned, for most young pre-presbyopic patients, preserving natural accommodation is much better than pursuing multifocality, not only from a medical standpoint but also from refractive one. For this, both laser vision correction (in suitable patients) and P-IOL implantation are excellent alternatives much better than multifocal pseudophakia. Pop and Payette [15] compared visual results and satisfaction of young patients implanted with Artisan phakic IOL (OPHTEC BV; Groningen, The Netherlands) with patients undergoing RLE finding that the former group had a better refractive prediction (were closer to emmetropia) and that P-IOL implantation “provided a better overall outcome for young patients with high hyperopia whose accommodation was preserved” [15]. It is crucial to keep in mind that this study included hyperopic patients, who have a decreased visual acuity not only for far vision but also for near vision. Highly myopic patients, tend to have excellent near vision (although their focal distance is short), so it is probable that satisfaction rates, especially for near vision, would be poor in case of multifocal IOL implantation.

Currently, most studies concerning pseudophakic IOL implantation in young pre-presbyopic patients include highly hyperopic cases. Nevertheless, for myopic patients, there is consensus that P-IOL implantation is way better alternative, as accommodation is preserved. The patient in this article was instructed to undergo RLE with a monofocal IOL implantation, allegedly to comply with Colombian social security laws, which cover phacoemulsification for cataracts, but do not cover P-IOL implantation. The patient claimed that the original idea of the prior surgeon was to perform the same surgery in the other eye and then proceed with, reading glasses for both eyes. This approach is, according to current knowledge, wrong and should be avoided if other options are available. As already discussed, performing phacoemulsification in young myopic patients has been linked to a higher likelihood of retinal detachment. Besides, if pseudophakia were necessary, as would be the case if the patient had a congenital cataract (which she did not have), it would have been better to implant a multifocal IOL to preserve some near vision which could help decrease symptoms, especially in the meantime before surgery of the other eye. When we met the patient, she had a great anisometropia and was highly symptomatic, especially for near vision. She did not tolerate her contact lens on her right eye and using glasses with a highly myopic prescription in only one eye was not tolerated and increased her visual strain.

Upon carefully discussing the case with the patient and her mother, we decided to perform P-IOL implantation in the contralateral eye. With this approach, we sought to correct her high myopia and anisometropia, while preserving accommodation so she could have usable vision for her near tasks. Implantation of posterior chamber P-IOL corrected myopia, and left the patient emmetrope, with excellent near and far vision, and complete resolution of her symptoms.

This case highlights the fact that correct decision making is of paramount importance when the surgeon is planning to correct ametropic patient, taking into account previous refractive state and age. Obviously, decisions should be based on the best clinical experience, and not solely on bureaucratic issues as seems to have been the case in the first surgery of the patient. In case of doubt, sound clinical judgment should guide decisions, and it should be kept in mind that the first obligation of the physician is to cause no harm to the patient, so sometimes it may be wiser to choose not to operate on patient if they cannot pay for the right surgical approach in their individual case.

This case also showed that, just as has been reported by Yasa et al. [16], implantation of a new posterior chamber P-IOL (EyeCryl Phakic Toric) is predictable and safe option for refractive correction in highly myopic eyes. Nevertheless, to date there are no published long term studies on this specific kind of lenses, so results, specially regarding endothelial cell stability and cataract formation, are currently lacking. It should be noted that both endothelial cell loss and cataract formation are among the described potential complications related to both anterior chamber and posterior chamber P-IOLs. In order to decrease the possibility of these complications, implantation of this kind of IOLs should be reserved for patients with a correctly-sized anterior chamber depth (of at least 2.8 mm as measured from corneal endothelium).

## 4. Conclusion

In the absence of clinically significant cataract, phacoemulsication in young pre-presbyopic high myopic patients may not be the best option in most scenarios. If it is to be done, implanting a multifocal IOL will probably be the best selection for most patients (if the macula is not compromised with myopic changes). Whenever it is possible, implantation of a P-IOL is a much better option, as it preserves natural accommodation, which is unparalleled by current pseudophakic IOLs, and in addition is not related with an increased risk of retinal detachment. This case also supports the excellent short-term refractive predictability and safety of Toric EyeCryl Phakic IOL, however long- term follow-up examinations of these patients are necessary in order to evaluate endothelial cells density and the transparency of the crystalline lens.
